# 

**Published:** 1894-07-14

**Authors:** 


**Notices of Births, Deaths, and Marriages** are inserted in this column at a charge of 2s. 6d. for an announcement not exceeding four lines.

**Notice to Advertisers and Subscribers.**

All Advertisements, Orders for Copies of The Hospital, and Business Communications should be addressed to **The Publisher, NOT TO THE EDITOR,** The Hospital, 428, Strand, W.C.

The Cost of Subscription, post free, is as follows:—Per week, 2½d.; per quarter, 2s. 8d.; per half-year, 5s. 3d.; per annum, 10s. 6d Foreign:—Per Annum, 12s. 8d.; payable, in all cases, in advance.

### NOTICE TO CORRESPONDENTS.

All MS., Letters, Books for Review, and other matters intended for the Editor should be addressed The Editor, The Lodge, Porchester Square, London, W.

The Editor cannot undertake to return rejected MS., even when accompanied by stamped directed envelope.

**"Burdett's Hospital Annual," 1894.**

Fifth year of publication. 900 pp. Boards, gilt lettering. A most complete guide to British, Colonial, Indian, and American Hospitals, Dispensaries, Nursing and Convalescent Institutions, and Asylums. Price 5s. Published by The Scientific Press (Limited), 428, Strand, W.C.

**NOTICE.—The Index for Vol. XV. (ending March 31st, 1894) is on sale at the Office of this Journal, price 2½d., post free.**

### SCRAPS AND GLEANINGS.

Burton Infirmary has received the munificent gift of £10,000 from Mr. W. H. Worthington.

Mr. George Hanbury has given £500 towards the building fund of the Paddington Green Children's Hospital.

A two-days' American fair has been held at Loughborough in aid of the funds of the Charwood Forest Convalescent Home.

The University of Halle will celebrate its second centenary on August 2nd of this year, when for three days the town will be *en fete*.

The Women's Medical College of the New York Infirmary held its 26th annual commencement this month, there being a class of seventeen lady students.

An "Infant Prince" Fund, in commemoration of the event, has been started at the Victoria Hospital for Children, S.W. Several contributions have already been promised.

The annual fête in aid of the Children's Hospital at Dublin has just taken place. This institution, St. Joseph's, was the first children's hospital which was established in Dublin.

The designs of Mr. Daniell Arkell have been accepted by the King's Norton Board of Guardians for the new infirmary at Selly Oak. The accommodation is intended for 250 inmates.

National Orthopædic Hospital.—On and after July 19th attendance will be given for out-patients at the hospital on the evening of Thursday, from half-past six to half-past seven o'clock.

A Chicago merchant has made a gift of 50,000 dollars to the Medical Department of the North-Western University to found a new professorship. The gift is made in honour of a medical friend, Dr. N. S. Davis.

The annual report of the Meath Hospital for the last year shows that that excellent institution is doing its efficient and charitable work under the most satisfactory conditions. The hospital has £334 to its credit.

The annual general report of the Hahnmann Free Dispensary shows that during the five and a-half years of its existence, 43,337 attendances have been registered. The accounts show a small balance to the good in hand.

The annual Hospital Sunday collection, which took place at Birmingham on Saturday, has resulted so far in the receipt of £9,900. The street collection, owing to the bad weather, shows a falling off as compared with last year.

The Royal National Consumption Hospital at Ventnor has benefited by a contribution of £2,000 for the cost of one house at the hospital. The donor has given this sum anonymously, in memory of two daughters who died from consumption.

An anonymous donor has sent £1,300 to the Swansea Hospital for the endowment of a bed at that hospital, and it is to be hoped that this will stimulate other local philanthropic people to come forward and assist in a work which commends itself to all.

At the seventy-sixth annual meeting of subscribers of the Manchester and Salford Lock Hospital. which took place last month, and to which reference has already been made, show the working expenses of the charity to be kept well within the income.

The financial and most important part of the scheme to build an Eye and Ear Hospital for the counties of Londonderry, Donegal, and Tyrone has developed wonderfully, and a collection taken up last week in the streets of the former city has ensured its success.

An especial appeal for funds for the North London Hospital for Consumption has been made by Mr. Hoare, L.C.C., treasurer to the hospital, in order to pay off the mortgage and building debt of £9,000, and to maintain the increased number of eighty beds.

A three days' "fancy fair," under the immediate patronage of Lady Emily Hart Dyke, was opened at the Sidcup Town Hall recently; the object of the fair being to clear a debt of £250 remaining on the purchase of some extra land for the benefit of the hospital at Sidcup.

The Society of the Curates' Augmentation Fund met at 2, Dean's Yard, Westminster, this week, the Venerable the Archdeacon of London presiding. The Council, whilst reporting a good year's work, earnestly appeal for increased income to meet the many urgent and pressing applications.

The annual meeting of subscribers of the Crewkerne Hospital Somersetshire, shows that the good work hitherto carried on has been maintained during the past year. By the liberality of Mr. J. Nicolls, jun., a concrete floor, with wooden blocks, has been provided for the out-patients' room.

An important meeting of the Warrington Infirmary Committee discloses a state of finances far from satisfactory to its managers. The committee are met face to face with a deficit of £1,266. To meet this difficulty Mrs. Beamont has already contributed £100, as has also Mr. Charterer Parr.

The Duke of Fife presided at a meeting at the Mansion House on Thursday, July 5th, on behalf of the Homes for Working Boys in London, the speakers being the Ven. Archdeacon Farrar and the Right Hon. A. J. Balfour, M.P. The chairman will be supported by the Earl of Meath, Lord Kinnaird, &c.

In the report of the Montrose Asylum House Committee it was stated the population had increased considerably during the past year, the average number resident having been 570 as against 552 in the previous year. The treasurer's statement for the year showed a surplus in favour of the managers of £1,439.

The lease of the Convalescent Home at Folkestone expires this year. The immediate necessity for new and extended premises is of a most earnest character. The total estimated cost of the undertaking is £3,250, on a site on the East Cliff overlooking the sea. Subscriptions and donations towards this much-needed charity are now solicited.

A dance in aid of the West London Hospital, held last week in the Queen's Hall, Langham Place, was a great success. More than 600 people were present, among whom were many old friends of the hospital. The string band of the Scots' Guards gave excellent music. We hope the institution will greatly benefit in funds by the entertainment.

### THE STUDENT OF MEDICINE.

### APPOINTMENTS.

Dr. Beasley appointed Medical Officer of Health to the Rowley Regis Local Board. H. A. Bryant, L.R.C.P.Lond., M.R.C.S.Eng., appointed House Surgeon to the Durham County Hospital. W. K. Bullmore, M.D.St.And., M.R.C.S.Eng., reappointed Medical Officer of Health to the Falmouth and Truro Port Sanitary Authority. Dr. Goodfellow reappointed Medical Officer of Health to the Brampton Local Board. R. Jones, M.D., C.M.Edin., D.P.H.Camb., appointed Health Officer and Compiler of Vital Statistics by the Merioneth County Council. W. W. Lake, M.R.C.S.Eng., reappointed Medical Officer of Health to the Guildford Rural Sanitary Authority. J. Maclaren, M.B., C.M.Edin., appointed Fifth Assistant Medical Officer to the West Riding Asylum, Wadsley, Sheffield. F. Tyndall, M.R.C.S.Eng., appointed Medical Officer to the Lostock Industrial School, vice G. Howarth, L.R.C.P.Edin., M.R.C.S.Eng., deceased. G. H. Williams, M.D.Yale, M.R.C.S.Eng., L.R.C.P.Edin., appointed Honorary Surgeon to Highland Hospital, Matteawan, New York.

### VACANCIES.

The following vacancies are announced this week, the amount of salary in each case being given after the name of the institution:

### Resident Medical Officers.

Cambridge Lunatic Asylum, Fulbourn, near Cambridge.—Assistant Medical Officer. Salary £140 per annum, with board, lodgings, and attendance in the Asylum. Applications to T. Musgrave Francis, Clerk to the Visitors, by July 21st.

East London Hospital for Children and Dispensary for Women, Glamis Road, Shadwell, E.—Resident Medical Officer. Must be registered practitioner in medicine and surgery. Salary £80 per annum, with board and residence. Applications and testimonials to Thomas Hayes, Secretary, by July 23rd.

Hospital for Consumption and Diseases of the Chest, Brompton.—Two Assistant Physicians and Resident House Physicians. Applications to William H. Theobald, Secretary, by July 18th and July 19th respectively.

North Staffordshire Infirmary and Eye Hospital, Hartshill, Stoke-upon-Trent.—House Physician. Salary £100 per annum, increasing £10 per annum at the discretion of the committee, with furnished apartments, board, and washing. Applications to the Secretary by July 23rd.

Liverpool Northern Hospital.—Assistant House Surgeon; doubly qualified. Salary £70 per annum, with residence and maintenance in the house. Applications to the Chairman by July 17th.

Royal London Ophthalmic Hospital, Moorfields, E.C.—Senior House Surgeon. The Junior House Surgeon is a candidate, and applicants are requested to state whether, in the event of his being appointed, they will be willing to accept the office of Junior. Salary £75 per annum for the Senior; Junior, £50 per annum, both with board and residence. Applications to the Secretary by July 19th.

University College, London.—Resident Medical Officer. Applications to J. M. Horsburgh, M.A., Secretary, by July 16th.

### Non-Resident Medical Officers.

National Dental Hospital, Great Portland Street, W.—Anæsthetist. Applications to E. Almack, Secretary, by July 19th.

### PASS LISTS OF THE UNIVERSITIES AND COLLEGES.

### University of Cambridge.

DEGREES.—At the Congregation on June 11th the following degrees in Medicine and Surgery were conferred: M.D.: D. W. Samways, M.A., late Fellow of St. John's; C. A. R. Sutton, M.A., Clare. M.B. and B.C.: F. E. A. Colby, B.A., King's; W. G. Peck, B.A., Trinity; H. M. Tickell, B.A., Trinity; H. B. Hewitt, B.A., Clare; C. C. Webb, B.A., Clare; F. N. Day, B.A., Caius; W. J. Harris, B.A., Caius; C. E. Sparks, B.A., Caius.

FIRST EXAMINATION FOR MEDICAL AND SURGICAL DEGREES, Easter Term, 1894. Part II: Elementary Biology.—Alder, Pemb.; Barnicot, Pemb.; T. W. Bates, M.A., Queens'; Joh. Bentley; Blyth, Trin. H.; G. W. Bond, Cla.; W. V. Braddon, Trin. H.; Brailey, Queens'; Brenan, Trin.; Briscoe, Pemb.; Bull, B.A., Gonv. and Cai.; Burfield, non-coll.; Burnand, Jes.; Chapman, Cla.; Child, Chr.; J. S. Clarke, Gonv. and Cai.; Coleman, Trin.; Davies, Gonv. and Cai.; Dunne, B.A., Queens'; W. Evans, Cla.; Foster, King's; Frend, Trin.; Fryer, Chr.; Gaitskell, Cla.; A. W. Greig, Jes.; Henderson, Gonv. and Cai.; Hopper, Gonv. and Cai.; Howitt, Joh.; Izard, Trin.; Jackson, Gonv. and Cai.; Killick, Trin.; Killick, Down.; J. M. E. Langton, Trin.; J. L. Lock, Gonv. and Cai.; P. G. Lock, Gonv. and Cai.; Lucas, Pemb.; McBryde, King's; McCaskie, Gonv. and Cai.; A. E. Martin, Down; Martineau, Emm.; Maxwell, Trin.; Morgan, Joh.; Mummery, Gonv. and Cai.; Nixon, Gonv. and Gai.; Parker, Emm.; Pearson, Emm.; Pearson, Sid Suss.; Pennington, Gonv. and Cai.; Percival, Joh.; Philbrick, Trin.; Ross, non-coll.; Rudman, King's; Salaman, Trin. H.; R. E. Sedgwick, Gonv. and Cai.; Shufflebotham, Trin.; Slack, Pemb.; Staynes, Chr.; Susmann, Gonv. and Cai.; J. G. Taylor, King's; E. C. Taylor, Joh.; T. P. Thomas, Gonv. and Cai.; Ticehurst, Cla.; Tyler, Joh.; Topham,

### THE STUDENT OF MEDICINE—*continued.*

Chr.; Walker, King's; Walker, Cla.; D. P. Watson, Trin.; West, Chr.; Wilkin, B.A. Pemb.; G. R. Wilson, Trin.; Wisdom, Emm.; Worthington, Trin.

Second Examination for Medical and Surgical Degrees, Easter Term, 1894. Part I: Pharmaceutical Chemistry.—Barham, Gonv. and Cai.; Boucher, Trin.; Brincker, Joh.; Browse, Cla.; E. H. Coleman, Joh.; H. St. C. Elliott, Trin.: W. R. Elliott, B.A. Joh. Ellis, Pet.: Fraser, Gonv. and Cai.; Green, B.A., Down.; Gutch, B.A., Chr.; Harwood, Trin.; Home, Trin.; Horne, B.A., Trin.; H. G. T. Jones, Joh., Keeling, Gonv. and Cai.; Letchworth, Emm.; H. R. Mayo, Gonv. and Cai.; Mills, B.A., Cla.; Mullings, Chr.; Naish, B A., Trin.; Nelson, B.A., Cla.; G. B. Nicholson, Cla.; Orton, Trin.; Perkins, B.A., Joh.; Reissmann, Joh.; Rowland, Down.; Sanderson, B.A., Cla.; Slade, B.A., Trin.; Stabb, Down.; Stanham, B.A., Trin.; Stawell, B.A., Trin. H.; Studd, Trin.; Sugden, Gonv. and Cai.; A. Thompson, B.A., Trin.; Turnbull, B.A., Gonv. and Cai.; Wingate-Saul, Trin. Part II: Human Anatomy and Physiology.—Barraclough, B.A., Chr.; Bedford, B.A., Trin.; Blatchford, B.A., Sidd. Suss.; Briggs, B.A., Joh.; Brown, B.A., Pemb.; Butler, Joh.; A. B. Carter, B.A., Jes.; A. E. Elliott, B.A., Joh.; Green, B.A., Down.; Jephcott, B.A., Gonv. and Cai.; Kirk, B.A., Chr.; McCleary, B.A., Trin. H.; Moritz, B.A., Gonv. and Cai.; Ogilvie, B.A., Chr.; Paterson, B.A., Emm.; Roe, Pemb.; Rowland, B.A., Down.; Sandilands, B.A., Trin.; Stabb, B.A., Down.; B. N. Tebbs, Queens'.; Tyrrell, B.A., Cla.; Wakefield, M.A., Trin.; F. K. Weaver, B A., Trin.; Williamson, B.A., Joh.; E. J. Woolley, B.A., Gonv. and Cai.

First Examination for M.B. and B.C., Easter Term, 1894. Chemistry and Physics.—Alder, Pemb.; S. Bennett, Down; Bentley, Emm.; W. V. Braddon, Trin. H.; W. P. S. Branson, Trin.; Brenan, Trin.; Brooke, Pemb.; Bull, B.A., Cai.; Burfield, non-coll.; Burnand, Jes.; Cheadle, Cai.: E. M. Clark, Trin.; Coleman, Trin.; J. G. Cooke, Sid. Suss.; Davies, Cai.; W. Evans, Cla.; Foster, King's; Fox, Emm.; Fraser, Jes.; Fryer, Chr.; Fuge, B.A., Sel.; Glenn, Pemb.; Greig, Jes.; Hawkins, Emm.; Hay, Cai.; Howitt, Joh.; Izard, Trin.; Killick, Down.; P. G. Lock, Cai.; McCaskie, Cai.; Martineau, Emm.; Marwell, Trin.; Mickletthwait, Trin.; Murison, B.A., Trin.; Nixon, Cai.; Orme, Cai.; Paterson, Cai.; Pearson, Sid. Suss.; Philbrick, Trin.; Pitkin, Ayerst's; Reynolds, Trin.; Roberts, Cla.; Ross, non-coll.; Rudman, King's; Saloman, Trin. H.; Shreiner, Down; R. E. Sedgwick, Cai.; Seyfang, Pet.; Sharples, Cai.; Shufflebotham, Trin.; Style, Emm.; Susmann, Cai.; T. P. Thomas, Cai.; Tyler, Joh.; Walker, Cla.; Ware, Pemb.; D. P. Watson, Trin.; A. G. Wilson, Cai.; T. Wood, Cai.

St. John's College.—Mr. F. Villy, B.A., scholar of St. John's College, has been elected to the Hutchinson Studentship for Research in Pathology.

Appointments.—The following appointments in the department of Natural Science and Medicine are announced: Mr. A. E. Shipley, Christ's, to be University Lecturer in Invertebrate Morphology; Mr. S. Ruhemann, Caius, to be University Lecturer in Organic Chemistry; Mr. J. J. Lister, St. John's, to be University Demonstrator of Comparative Anatomy.

